# Isolated Adrenocorticotropic Hormone Deficiency Diagnosed After the Cessation of Glucocorticoid Therapy for Eosinophilic Esophagitis: A Case Report

**DOI:** 10.7759/cureus.66217

**Published:** 2024-08-05

**Authors:** Shogo Iwamura, Shiori Watts, Kazuma Sakuraba, Yohei Yamamoto, Daisuke Matsuda

**Affiliations:** 1 Division of Diabetes and Endocrinology, Nakadori General Hospital, Akita, JPN; 2 Division of Neurology, Nakadori General Hospital, Akita, JPN; 3 Division of Gastroenterological Surgery, Nakadori General Hospital, Akita, JPN; 4 Division of Pathology, Nakadori General Hospital, Akita, JPN

**Keywords:** patient management, endocrine diseases, glucocorticoid therapy, eosinophilic esophagitis, adrenal insufficiency, isolated adrenocorticotropic hormone deficiency

## Abstract

Isolated adrenocorticotropic hormone deficiency (IAD) is a rare pituitary disorder that can cause adrenal insufficiency. However, due to its nonspecific symptoms, its diagnosis is often difficult and may be delayed. Patients with IAD require lifelong glucocorticoid (GC) replacement therapy. Contrastingly, GC-induced secondary adrenal insufficiency is a reversible condition that arises when patients receiving GC therapy reduce their GC dosage or discontinue therapy. Differentiating between IAD and GC-induced secondary adrenal insufficiency is clinically crucial. We report a unique case that required differentiation between these two conditions.

A 71-year-old Japanese woman presented with symptoms of adrenal insufficiency after discontinuation of GC therapy for eosinophilic esophagitis. We conducted detailed interviews and repeated the endocrinological examinations. We concluded that her symptoms were owing to IAD rather than GC-induced secondary adrenal insufficiency.

She began a lifelong hydrocortisone replacement therapy. This case suggests that when caring for patients undergoing GC therapy, it is important to consider the possibility of coexisting IAD and arrange endocrinological examinations if signs of adrenal insufficiency arise during the gradual reduction of GC treatment.

## Introduction

Isolated adrenocorticotropic hormone deficiency (IAD) is a relatively rare pituitary disorder of unknown etiology with reported prevalence rates of 1.9-7.3 cases per 1,000,000 in Japan [1]. It leads to secondary adrenal insufficiency (SAI) and presents with nonspecific symptoms such as generalized fatigue, loss of appetite, and weight loss, often causing delays in diagnosis [2]. Moreover, eosinophilic esophagitis (EoE) is a chronic inflammatory disease of the esophagus mediated by antigens, with the most frequently studied factors being food allergens, and its prevalence has steadily increased in recent years [3,4]. The treatment options for EoE can include proton pump inhibitors, corticosteroids, and biologics [4].

Herein, we present a case of IAD diagnosed after the cessation of glucocorticoid (GC) therapy for EoE. The clinical course was complicated by masking of IAD symptoms with GC therapy for EoE. The diagnosis of IAD was established through meticulous interviews and endocrinological assessments.

## Case presentation

**Figure 1:**
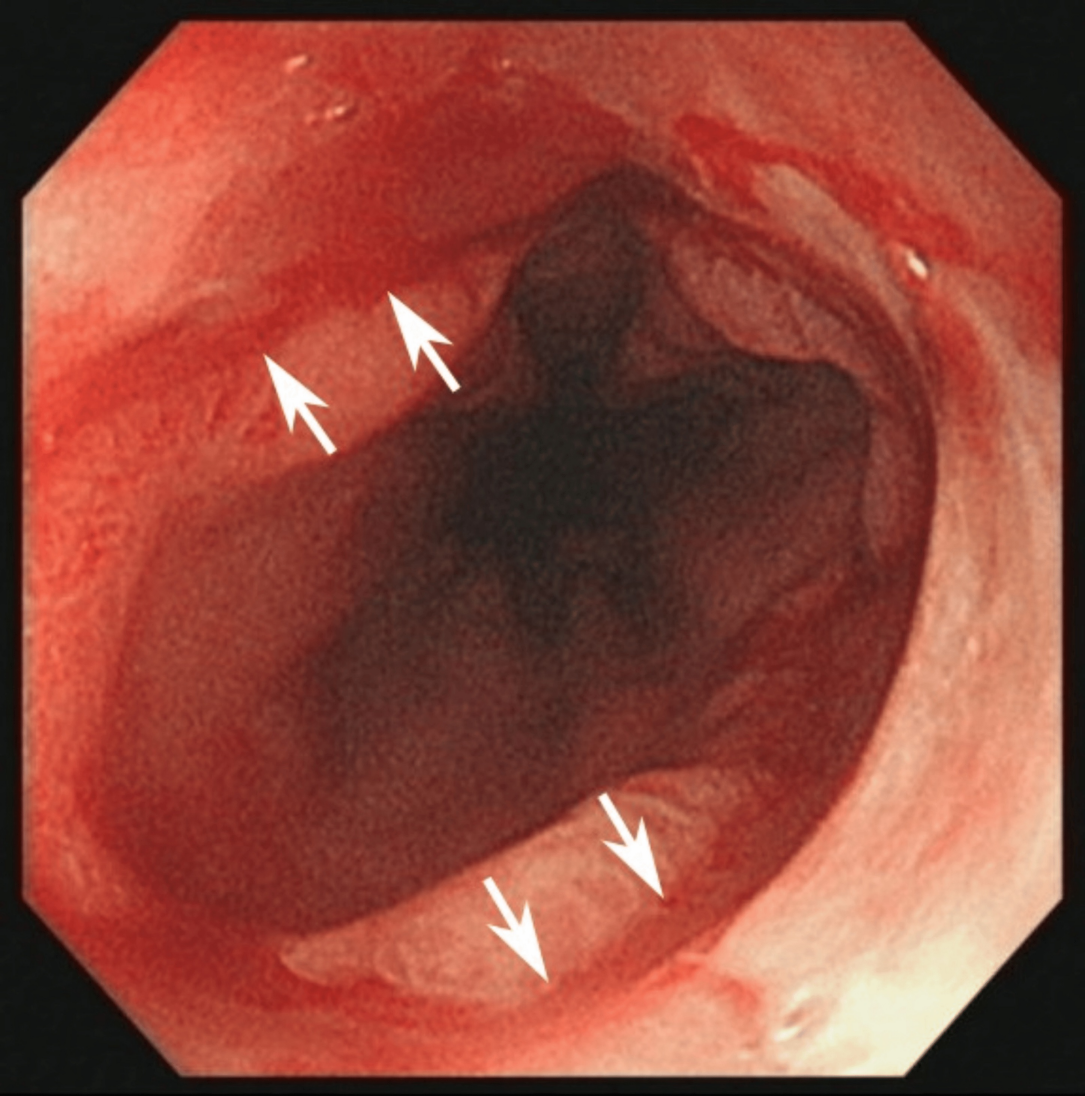
EGD performed in November 2022. EGD findings showed an esophageal ulcer (white arrows) and severe inflammation of the mucosa. EGD: esophagogastroduodenoscopy.

**Figure 2:**
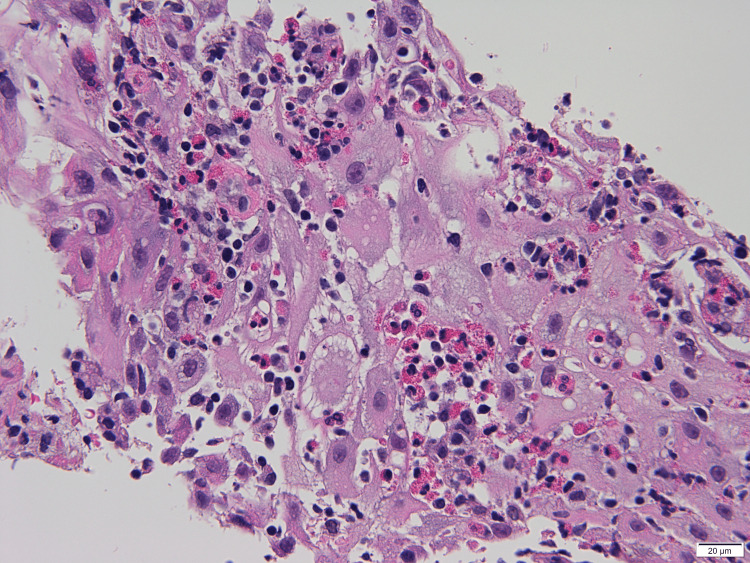
Histopathological findings of the biopsied esophageal tissue (November 2022). Notable infiltration of eosinophils was observed, indicating eosinophilic esophagitis (hematoxylin and eosin staining).

A 71-year-old Japanese woman presented with fatigue, loss of appetite, nausea, dysphagia, and precordial pain that gradually began in early October 2022 and prompted her to seek medical attention at a local clinic. Her medical history included ovarian cyst surgery. Her mother had a family history of thyroid surgery. However, no other endocrine disorders were reported. She had no history of smoking or alcohol consumption. Esophagogastroduodenoscopy (EGD) was performed at a local clinic, raising the suspicion of esophageal candidiasis, for which she was prescribed oral miconazole. However, her symptoms did not improve and she was referred to our hospital. Upon admission, EGD and endoscopic biopsy were conducted, which resulted in a diagnosis of EoE (Figures 1, 2). Treatment commenced with oral vonoprazan (20 mg/day) and topical budesonide therapy (800 μg/day). Nevertheless, as her symptoms showed minimal improvement, topical budesonide was discontinued six days after its initiation, and oral prednisolone (30 mg/day) was started, leading to rapid amelioration of her condition. The following week, topical budesonide therapy was reintroduced, and oral prednisolone was gradually tapered. The subsequent EGD indicated improvement in EoE; however, it also revealed a stricture that necessitated dilation of the esophagus. The patient was discharged from the hospital in late December after endoscopic dilation of the esophagus.

Oral prednisolone treatment was discontinued in January 2023. In late March 2023, topical budesonide therapy was also discontinued. After cessation of oral prednisolone, the patient experienced symptoms of bilateral shoulder-to-upper arm pain, general fatigue, and loss of appetite, which led to readmission in mid-May.

Low levels of adrenocorticotropic hormone (ACTH) and cortisol were observed; hence, the patient was transferred to our division.

According to a physical examination at her transfer, her consciousness was clear, and her body length, body weight, body mass index, blood pressure, and pulse rate were 148.0 cm, 38.0 kg, 17.3 kg/m^2^, 121/83 mmHg, and 84 beats per minutes, respectively. She presented with severe emaciation, weakness, and pain upon pressure in the muscles of the shoulders and upper extremities; however, no other neurological abnormalities were observed. No abnormal findings were observed on her chest, abdomen, or skin. Laboratory data showed low levels of plasma ACTH (< 1.5 pg/mL), serum cortisol (0.32 µg/dL), insulin-like growth factor-1 (12 ng/mL), total protein (5.3 g/dL), albumin (3.2 g/dL), glucose (65 mg/dL), white blood cells (2,620 /µL), hemoglobin (8.7 g/dL), and platelets (8.8×10^4^ /µL), and high levels of serum aspartate aminotransferase (59 IU/L), alanine aminotransferase (46 IU/L), alkaline phosphatase (114 IU/L), gamma-glutamyl transpeptidase (33 IU/L), C-reactive protein (0.25 mg/dL), thyroid-stimulating hormone (TSH) (10.79 µIU/mL), growth hormone (GH) (10.4 ng/mL), prolactin (PRL) (67.8 ng/mL), and arginine vasopressin (7.9 pg/mL) (Table 1).

**Table 1:** Laboratory findings on transfer (June 2023). Blood samples were collected in the morning (9 am) with the patient in the supine position.

Parameter	Result	Reference range
Hematology		
Red blood cells	294×10^4^/µL	386-492
Hemoglobin	8.7 g/dL	11.6-14.8
Hematocrit	25.60%	35.1-44.4
White blood cells	2,620/µL	3,300-8,600
Platelets	8.8×10^4^/µL	15.8-34.8
Blood chemistry		
Total protein	5.3 g/dL	6.6-8.1
Albumin	3.2 g/dL	4.1-5.1
Aspartate aminotransferase	59 IU/L	13-30
Alanine aminotransferase	46 IU/L	7-23
Alkaline phosphatase	114 IU/L	38-113
Gamma-glutamyl transpeptidase	33 IU/L	9-32
Urea nitrogen	16.9 mg/dL	8-20
Creatinine	0.49 mg/dL	0.46-0.79
Sodium	140 mEq/L	138-145
Potassium	3.8 mEq/L	3.6-4.8
Chloride	104 mEq/L	101-108
C-reactive protein	0.25 mg/dL	0-0.14
Immunoglobulin G4	14.1 mg/dL	11-121
Thyroid peroxidase antibody	4.7 IU/mL	<3.3
Thyroglobulin antibody	1,310 IU/mL	<19.3
Fasting plasma glucose	65 mg/dL	70-109
Glycated hemoglobin	4.80%	4.6-6.2
Endocrinology		
Adrenocorticotropic hormone	<1.5 pg/mL	7.2-63.3
Cortisol	0.32 µg/dL	6.24-18.00
Thyroid-stimulating hormone	10.79 µIU/mL	0.61-4.23
Free thyroxine	0.76 ng/dL	0.71-1.52
Free triiodothyronine	2.51 pg/mL	2.39-4.06
Growth hormone	10.4 ng/mL	0.13-9.88
Insulin-like growth factor-1	12 ng/mL	56-172
Luteinizing hormone	22.87 mIU/mL	5.72-64.31
Follicle-stimulating hormone	44.42 mIU/mL	<157.79
Progesterone	<0.05 ng/mL	<0.33
Estradiol	<5.0 pg/mL	<47.0
Prolactin	67.8 ng/mL	3.12-15.39
Arginine vasopressin	7.9 pg/mL	<2.8

**Figure 3:**
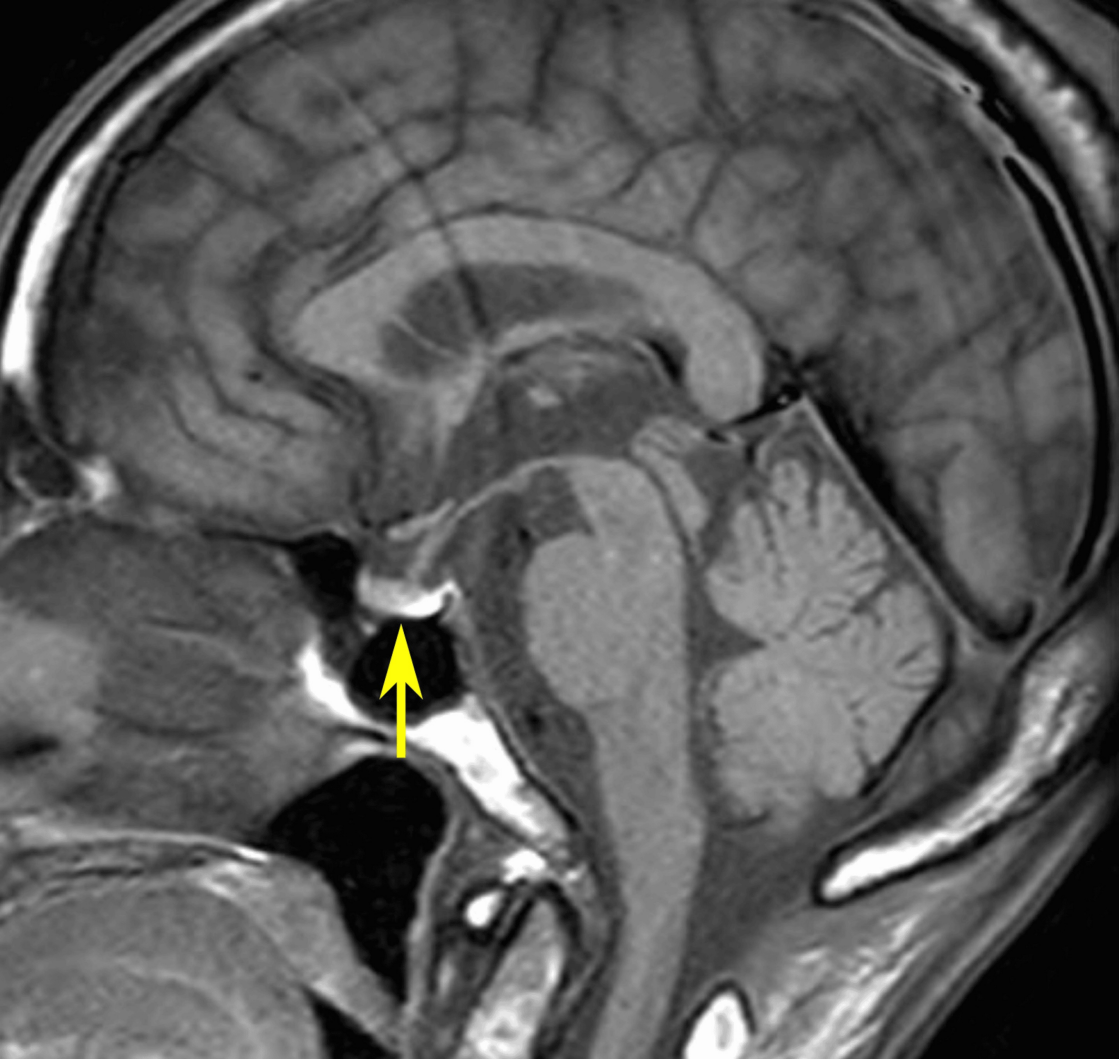
Brain MRI performed in June 2023. Sagittal T1-weighted plain MRI showed no abnormality in the pituitary gland (yellow arrow). MRI: magnetic resonance imaging.

She tested positive for thyroid peroxidase antibody (4.7 IU/mL) and thyroglobulin antibody (1,310 IU/mL). Dynamic tests assessing pituitary hormone secretion revealed normal release of TSH, GH, and PRL, with age-appropriate release of luteinizing hormone and follicle-stimulating hormone. However, ACTH release was notably absent upon stimulation with corticotropin-releasing hormone (CRH) (Tables 2, 3). Decreased cortisol response was observed during the rapid ACTH stimulation test (Table 4). In the insulin tolerance test, no response of ACTH or cortisol was observed (Table 5). There were no apparent abnormalities on brain magnetic resonance imaging (Figure 3). Computed tomography of the chest and abdomen revealed no abnormalities in the heart, lungs, liver, spleen, pancreas, kidneys, or adrenal glands.

**Table 2:** CRH/TRH/LHRH stimulation test conducted in June 2023. The following were injected intravenously in the morning (9 am): human corticotropin-releasing hormone (CRH, 100 µg), thyrotropin-releasing hormone (TRH, 200 µg), and luteinizing hormone-releasing hormone (LHRH, 100 µg). N.M.: not measured.

Parameter	Time (min)			
	0	30	60	90
Adrenocorticotropic hormone (pg/mL)	<1.5	<1.5	<1.5	N.M.
Cortisol (µg/dL)	0.33	N.M.	0.34	0.32
Thyroid-stimulating hormone (µIU/mL)	9.72	57.93	52.30	N.M.
Prolactin (ng/mL)	57.8	201.0	178.0	N.M.
Luteinizing hormone (mIU/mL)	27.33	53.94	66.37	N.M.
Follicle-stimulating hormone (mIU/mL)	46.59	N.M.	60.81	66.80

**Table 3:** GHRP-2 stimulation test conducted in June 2023. Growth hormone-releasing peptide-2 (GHRP-2, 100 µg) was injected intravenously in the morning (9 am).

Parameter	Time (min)				
	0	15	30	45	60
Growth hormone (ng/mL)	9.5	38.4	41.8	34.7	27.3

**Table 4:** Rapid ACTH stimulation test conducted in June 2023. Synthetic adrenocorticotropic hormone (ACTH) 1-24 (tetracosactide acetate 0.25 mg) was intravenously injected in the morning (9 am).

Parameter	Time (min)		
	0	30	60
Cortisol (µg/dL)	0.25	2.24	3.26

**Table 5:** Insulin tolerance test conducted in June 2023. Human insulin (four units) was injected intravenously in the morning (9 am). N.M.: not measured.

Parameter	Time (min)			
	0	30	60	90
Plasma glucose (mg/dL)	85	38	49	51
Adrenocorticotropic hormone (pg/mL)	<1.5	<1.5	<1.5	N.M.
Cortisol (µg/dL)	0.31	N.M.	0.59	0.59

Based on these findings, the patient was diagnosed with SAI. She started taking 15 mg/day of hydrocortisone orally, and her fatigue, loss of appetite, and pain in the shoulders and upper limbs improved rapidly. On day 42 after the transfer, the patient was discharged. As her symptoms began several weeks after the discontinuation of oral prednisolone, it was uncertain whether her SAI stemmed from GC-induced SAI or pre-existing IAD. In mid-November 2023, four months post discharge, a rapid ACTH stimulation test and CRH stimulation test were conducted to confirm the diagnosis. During these tests, hydrocortisone (10 mg/day) was administered orally. In the rapid ACTH stimulation test, the cortisol response decreased (Table 6), whereas ACTH release was absent in the CRH stimulation test (Table 7).

**Table 6:** Rapid ACTH stimulation test performed in November 2023. Synthetic adrenocorticotropic hormone (ACTH) 1-24 (tetracosactide acetate 0.25 mg) was intravenously injected in the morning (9 am).

Parameter	Time (min)		
	0	30	60
Cortisol (µg/dL)	0.15	2.22	2.92

**Table 7:** CRH stimulation test conducted in November 2023. Human corticotropin-releasing hormone (CRH, 100 µg) was injected intravenously in the morning (9 am). N.M.: not measured.

Parameter	Time (min)			
	0	30	60	90
Adrenocorticotropic hormone (pg/mL)	<1.5	<1.5	<1.5	N.M.
Cortisol (µg/dL)	0.19	N.M.	0.18	0.18

These findings were similar to the initial stimulation test results, supporting a diagnosis of IAD. When reviewing her medical history again, it was noted that she had easily become fatigued and had quit her job as a staff member at a grocery store because of her fatigue approximately one year before being diagnosed with EoE. This suggests the presence of adrenal insufficiency before EoE treatment. Therefore, we concluded that the patient developed IAD before the onset of EoE, and the symptoms of IAD became apparent in conjunction with the onset of EoE. Treatment with GCs for EoE unintentionally improved her symptoms of IAD; however, the cessation of GC therapy led to the recurrence of IAD symptoms. After the stimulation tests, hydrocortisone replacement therapy was continued. During outpatient follow-up, no recurrence of symptoms was observed.

## Discussion

**Figure 4:**
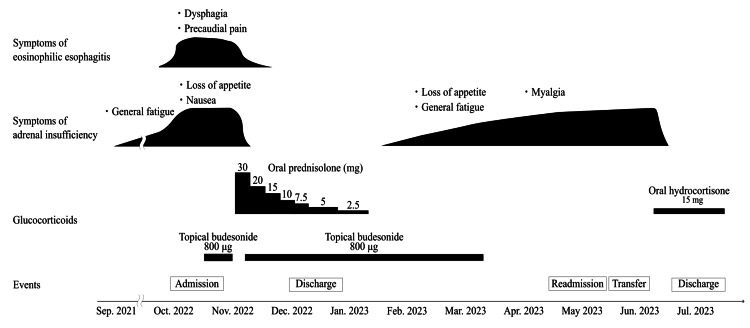
Clinical course. The use of glucocorticoids to treat eosinophilic esophagitis unintentionally improved symptoms of IAD. However, once oral prednisolone was stopped, the symptoms reappeared. After the diagnosis of IAD, initiation of oral hydrocortisone replacement therapy immediately ameliorated the symptoms. IAD: isolated adrenocorticotropic hormone deficiency.

We encountered a case of an older adult woman who was diagnosed with IAD after treatment for EoE (Figure 4). We concluded that the patient had pre-existing IAD before the onset of EoE. Symptoms of IAD became more evident after the onset of EoE and were inadvertently alleviated by GC therapy; however, the symptoms resurfaced upon cessation of the treatment and disappeared again with hydrocortisone replacement therapy.

This case highlights two important points. Firstly, it underscores the significance of distinguishing between GC-induced SAI and IAD in patients who have discontinued GC therapy. Secondly, it emphasizes the importance of meticulously assessing the clinical course during the differentiation process.

GCs are widely used owing to their anti-inflammatory and immunosuppressive properties. However, a notable drawback of GC therapy is the suppression of the hypothalamic-pituitary-adrenal (HPA) axis, potentially resulting in GC-induced SAI [5]. Daily administration of 20 mg or more of hydrocortisone or its equivalents for longer than three weeks may be associated with the suppression of ACTH secretion [6]. In this case, oral prednisolone treatment was administered for approximately two months and was discontinued several weeks before the onset of adrenal insufficiency symptoms. Therefore, it was necessary to differentiate between GC-induced SAI and IAD. If it had been GC-induced SAI, there was a possibility that the secretion of ACTH may have recovered after a certain period [5]. Typically, the HPA axis recovers within four weeks after discontinuing continuous GC usage [5], although in some instances, it may take longer than six months [7]. Therefore, we conducted stimulation tests eight months after the cessation of GC treatment for EoE. In this case, no signs of recovery of ACTH secretion were observed, leading to the diagnosis of IAD. Furthermore, IAD is an extremely rare condition. Conversely, numerous patients undergo GC therapy under various conditions, raising the possibility that some may have pre-existing IAD. Generally, if diseases treated with GCs improve and clinicians decide to taper them, they must plan for a gradual reduction in the GC dose to prevent GC-induced SAI. However, if the patient has IAD, the secretion of ACTH and cortisol never recovers, even with careful tapering. These patients must undergo GC replacement therapy for life. Therefore, this differentiation is crucial. It is essential to consider the possibility of IAD and schedule endocrinological investigations if symptoms of adrenal insufficiency emerge during the gradual tapering of GC therapy.

Furthermore, in understanding the clinical course of this case, it was crucial to carefully confirm the medical history of the patient. This allowed us to ascertain that symptoms of IAD were also present at the onset of EoE.

EoE is an antigen-mediated disease characterized by infiltration of eosinophils into all layers of the esophagus, leading to an inflammatory response. In adults, the manifestations are dysphagia (46.2-94.5%), food impaction (16.9-65.7%), and heartburn (7.7-54.5%) [4]. Adults demonstrate fewer symptoms typical of children such as nausea and vomiting [8].

Conversely, IAD is a rare endocrine disorder characterized by reduced or absent production of ACTH in the pituitary gland, leading to SAI. ACTH secretion is impaired in IAD, although other pituitary hormones are normally secreted and there are no structural abnormalities in the pituitary gland [9]. Moreover, patients with IAD typically exhibit vague symptoms, including fatigue, loss of appetite, unintentional weight reduction, and a tendency to develop hypoglycemia [5]. In this case, at the onset of EoE, the patient presented with local symptoms such as dysphagia and precordial pain, as well as systemic symptoms including fatigue, loss of appetite, and nausea. Systemic symptoms are relatively rare in patients with EoE. This suggests that the patient may have developed IAD before the onset of EoE. Furthermore, the medical history indicated increased fatigue approximately one year before the onset of EoE providing further evidence supporting this hypothesis.

In this unique case, the symptoms of pre-existing IAD were masked by GC therapy for EoE, and upon discontinuation of GC therapy, the symptoms reappeared. We confirmed the diagnosis by reassessing the patient’s medical history and repeating stimulation tests.

## Conclusions

We experienced a unique case of IAD masked by GC therapy for EoE. It was difficult to differentiate between IAD and GC-induced SAI, but we successfully diagnosed the patient according to meticulous history-taking and repeated stimulation tests.

Among patients with conditions requiring GC therapy, including EoE, there is a possibility of pre-existing IAD. Therefore, when symptoms of adrenal insufficiency appear upon gradual tapering or cessation of GCs, it is important to consider the possibility of IAD, reassess the clinical course, and plan endocrinological examinations accordingly.
